# Kinematics and directionality of body turning in water striders (*Gerris argentatus*) on the water surface

**DOI:** 10.1111/1744-7917.13486

**Published:** 2025-01-06

**Authors:** Javad Meshkani, Hamed Rajabi, Alexander Kovalev, Stanislav N. Gorb

**Affiliations:** ^1^ Functional Morphology and Biomechanics, Institute of Zoology Kiel University Kiel Germany; ^2^ Division of Mechanical Engineering and Design, School of Engineering London South Bank University London United Kingdom

**Keywords:** aquatic insects, biomechanics, Heteroptera, locomotion, rowing, surface tension

## Abstract

Water striders inhabit the elastic surface tension film of water, sharing their environment with other aquatic organisms. Their survival relies heavily on swift maneuverability and navigation around floating obstacles, which aids in the exploration of their habitat and in escaping from potential threats. Their high agility is strongly based on the ability to execute precise turns, enabling effective directional control. This paper investigates the intricate coordination of leg movements essential for initiating and sustaining turning maneuvers in water striders. We elucidate the distinct roles of each leg in modulating posture and stability during turns, with a focus on the pivotal role of the midlegs in maintaining directional movement. Through analysis of leg accelerations, decelerations, and load distribution, we unveil the spatiotemporal dynamics governing successful turns. Our findings reveal refined turning strategies employed by water striders in varying situations, from narrow to wide turns, characterized by adaptations in their locomotor system, particularly in the widening of the sculling field. Additionally, we report the phenomenon of reverse sculling, a novel escape tactic of water striders. By shedding light on the maneuverability of water striders, this study contributes to a deeper understanding of animal locomotion strategies in aquatic environments.

## Graphical abstract

This graphical abstract illustrates the kinematics and directionality of body turning in water striders (*Gerris argentatus*). The top image shows a water strider making turns, with arrows indicating possible directions and a dashed line tracing its path, highlighting its dynamic turning capabilities. The bottom image offers a top‐down view, showing points of contact and load distribution via leg shadows, emphasizing the water strider's position and movement.

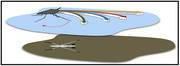

## Introduction

Water striders inhabit the water surface film alongside various other aquatic organisms (Andersen, 1982). This environment poses constant threats from predators both above and below the water surface (Haskins *et al.*, 1997; Krupa & Sih, 1999; Armisén *et al.*, 2015). To fulfill their essential tasks, such as foraging, mating, and avoiding predation, water striders engage in a wide array of locomotor behaviors (Andersen, 1976; Caponigro & Eriksen, 1976). Their survival, therefore, depends on possessing a high level of maneuverability at the air‐water interface.

The six‐legged stance represents the most inherently stable posture, offering a spectrum of maneuvering capabilities (Ting *et al.*, 1994; Raibert *et al.*, 1995; Gruhn *et al.*, 2009; Cao *et al.*, 2016; Weihmann *et al.*, 2017; Dürr *et al.*, 2019). While sprawled‐postured arthropods have been noted for their mechanical stability and agility (Full *et al.*, 1991; Ting *et al.*, 1994; Full & Koditschek, 1999; Jindrich & Full, 1999; Koditschek *et al.*, 2004; Ritzmann *et al.*, 2004; Chen *et al.*, 2006), specific aspects of maneuverability in water striding animals remain largely unexplored. Symmetrical leg movements are imperative for straightforward locomotion (Gruhn *et al.*, 2009), as evidenced by the tripod gait observed in walking insects (Fisher *et al.*, 2001; Cruse *et al.*, 2003; Bender *et al.*, 2011; Wu *et al.*, 2015; Barrio *et al.*, 2021; Chun *et al.*, 2021). Insects exhibit a wide range of gaits beyond the well‐known tripod gait (Wilson, 1966), including tetrapod (Graham, 1972), wave (Pelletier & Caissie, 2001), metachronal rhythm (Manton, 1952), caterpillar crawling (Brackenbury, 1999), cockroach running (Full & Tu, 1991), and water strider sliding (Hu *et al.*, 2003).

Smooth transitions between gaits and directional changes, such as curve walking, necessitate precise coordination of leg movements (Frantsevich & Gorb, 2004; Dürr & Ebeling, 2005; Billeschou *et al.*, 2021; Weihmann, 2022; Phodapol *et al.*, 2023). Insects leverage their multijointed exoskeletons to execute changes in direction and perform complex maneuvers, with each joint contributing its specific movement to achieve smooth motion (Nye & Ritzmann, 1992; Gorb, 1995; Frantsevich *et al.*, 1996; Frantsevich, 1998; Gruhn *et al.*, 2009; Larsen *et al.*, 2023). Each insect leg, comprised distinct segments, such as the coxa, trochanter, femur, tibia, and tarsus, plays a unique role in locomotion (Richards & Davies, 1977; Chapman, 1998). Specifically in water striders, the forelegs, midlegs, and hindlegs utilize the tarsus, terminal tarsus section, and tarsus‐tibia section, respectively, as points of contact with the water surface.

Insects must maintain balance not only while walking, but also during transitions, such as turning their bodies to change direction (Jander, 1985; Aminzare *et al.*, 2017). Water striders exhibit frequent adjustments in leg positioning throughout different stages of motion to optimize their motion trajectory (Lu *et al.*, 2018). Leveraging the surface tension of water, their legs serve multiple functions, including generating stroke power, providing support, stabilizing the body, and directing locomotion (Hu *et al.*, 2003; Bush & Hu, 2006; Zheng *et al.*, 2016; Lu *et al.*, 2018; Steinmann *et al.*, 2018; Meshkani *et al.*, 2023a). Studying water striders offers valuable insights into the kinetics underlying maneuverability in striding insects, contributing to a more cohesive understanding of their locomotory capabilities.

In this study, we primarily address three questions: (1) What turning patterns do water striders use? (2) How do leg movement patterns influence water strider turning angles? (3) Which factors contribute to achieving body stability during a turn? Here, we categorized turning modes of water striders into three distinct categories. This research may also potentially aid in the development of water‐walking machines capable of both linear motion and maneuverability.

## Materials and methods

### Experimental animals and preparations

Water striders (*Gerris argentatus*) were collected from a pond in the botanical garden of Kiel University and subsequently housed in aquaria maintained at room temperature (25 °C). In the laboratory, certain groups of insects underwent amputation procedures. We immobilized their legs using a bead of glue at the coxa‐femur joint, with the femur flexed upward to prevent wounding and misbalance from asymmetrical weight removal, ensuring the leg tip stayed clear of the water surface.

### Protocol of measurement of load changes

The methodology to estimate load changes on the legs of water striders utilizes the shadow tracking method, an approach validated and refined across several studies (Yin *et al.*, 2016; Zheng *et al.*, 2016; Lu *et al.*, 2018; Meshkani *et al.*, 2023a). In this procedure, as water striders sustain their stance atop the water film, their legs cause the formation of distinctive dimples on the water surface. These dimples alter the path of light passing through, creating shadow regions with a clearly visible bright perimeter on the aquarium floor (Yin *et al.*, 2016; Zheng *et al.*, 2016). By quantifying these shadow areas, researchers can accurately measure the loads applied to each leg (Zheng *et al.*, 2016). The shadows expand as the load increases and the dimple deepens, altering the water surface curvature and refracting light more significantly, thereby expanding the shadow area.

Several key relationships have been established to affirm the reliability of the shadow method: the correlation between the loading force and the shadow area (Zheng *et al.*, 2016); the congruence between the animal's body weight, as measured by an electronic balance, and the shadow area generated by the legs (Zheng *et al.*, 2016; Meshkani *et al.*, 2023a); the relationship between the force acting on a leg and the resultant depth of the dimple, reflected by the shadow area at the base of the vessel (Zheng *et al.*, 2016); and the associations linking the shadow area with the topography of the dimple (Lu *et al.*, 2018; Steinmann *et al.*, 2018).

Utilizing shadow data, the load applied to each leg during motion can be calculated, focusing on the relative load distribution expressed as a percentage of the total body weight of the animal. Moreover, the technique enables continuous tracking of the interaction points between the legs and the water surface, documented through high‐speed video analysis. This comprehensive approach not only quantifies load but also provides insights into the dynamic stability and propulsion mechanics of water striders.

### Experimental setup

**Fig. 1:**
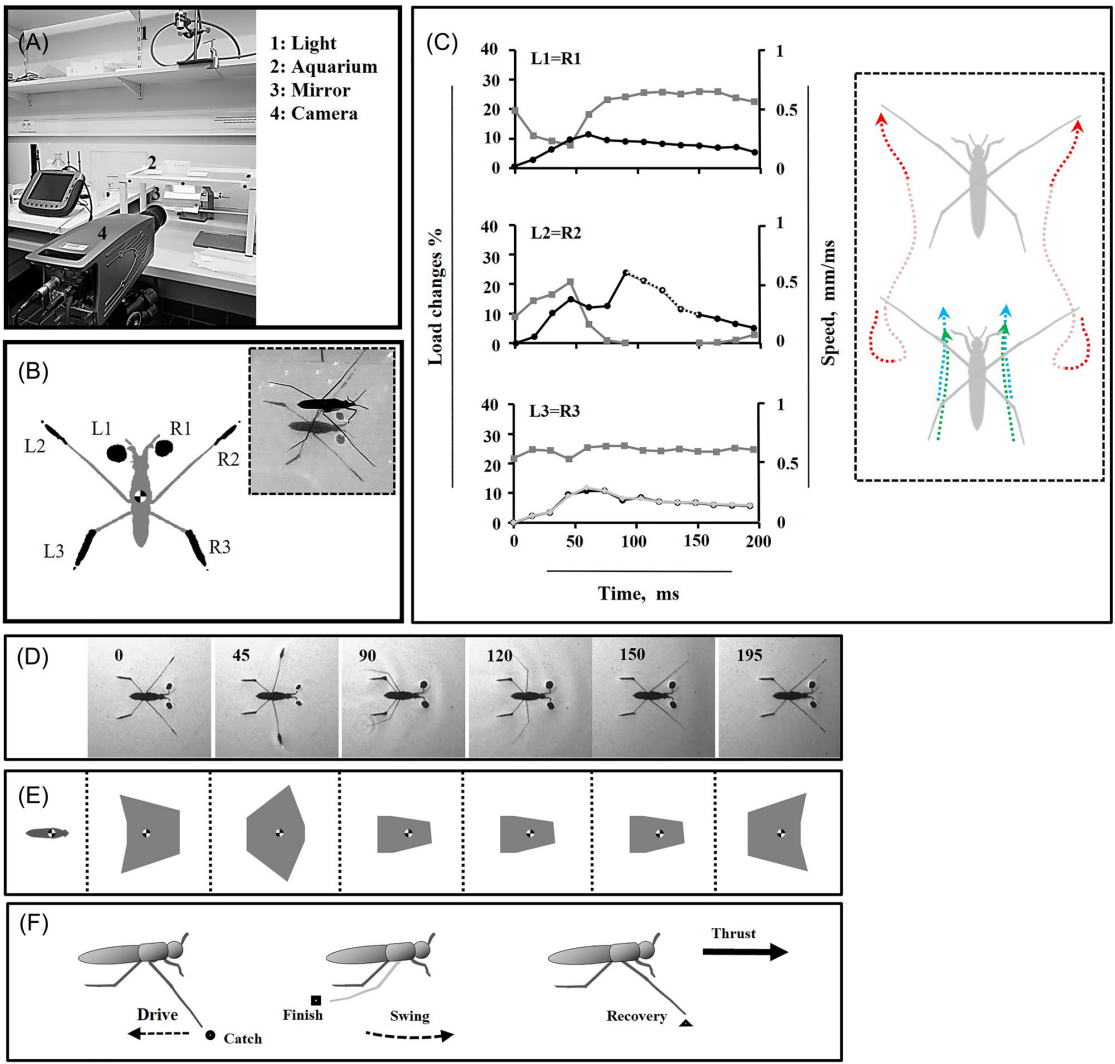
Kinematics of legs during forward striding. (A) The experimental setup. (B) Shadows produced by the foreleg, midleg and hindleg that are labeled with L1, L2 and L3 on the left side, and R1, R2 and R3 on the right side, respectively. The insect on the water surface and shadows on the aquarium floor (subplot B). (C) Load distribution on the legs (gray lines), speed of legs (black lines) along motion trajectory, and speed of tip of hindlegs (light gray) (since both lines have the same speed, they are overlapped), the dotted part of black line for L2 = R2 indicates the duration of the midleg in the air. The trajectory of the midlegs (red dotted lines), along with that of the hindlegs including the tips (green dotted lines) and the tibia‐femur joints (blue dotted lines) is depicted during motion in subplot (C). The faint segment of the red dotted line indicates the duration of the midleg in the air. (D) High‐speed sequence of shadows in a striding cycle. The number in each frame indicates the time in millisecond. (E) Schemes of corresponding BOS (gray areas), corresponding to the single frames in (D), during striding. (F) Schematic of a sculling cycle, the side view. The circle, square and triangle indicate three key positions of the midlegs at catch, finish and recovery points, respectively. The straight dashed‐arrow line indicates backward motion of the midlegs; the curved dashed‐arrow line indicates forward motion of midlegs.

Water striders (*N* = 3 per measurement) were observed traversing a vessel with dimensions of 10 cm length, 5 cm width, and 5 cm height, filled with distilled water to a height of 5 mm (Fig. 1A). A Storz Techno Light 270 Cold Light Projector was positioned above the vessel to provide sufficient illumination, and to enhance visibility, the bottom of the vessel was lined with a white semi‐transparent polymer sheet (GBC laminating films, 125 µm thick). An inclined mirror set at a 45° angle was situated beneath the vessel to capture shadow images. Each turning sequence was recorded using a high‐speed camera with a frame rate of 2000 frames per second (Olympus I‐Speed 3 Series High‐Speed Cameras). Motion sequences were allowed to occur naturally to facilitate the insect behavior and achieve the desired timing and angle of turning. All experiments were conducted at room temperature (25°C).

### Analysis procedure

Captured videos were analyzed frame‐by‐frame using ImageJ software to measure the area of shadows, following the method outlined by Schneider *et al.* (2012). Subsequently, the Manual Tracking plugin within ImageJ was utilized to track the positions of the body center and legs throughout the striding cycle. To provide a comprehensive understanding of leg kinematics, schematic illustrations were created. Statistical analyses were conducted using SigmaPlot 12.0 software (Systat Software Inc., San José, CA, USA).

### Symbols used

In our notation system, L1 represents the left foreleg, L2 the left midleg, L3 the left hindleg, R1 the right foreleg, R2 the right midleg, and R3 the right hindleg. To investigate the potential effects of leg ablation, we created amputated preparations with specific amputated legs. In this notation, “‐” signifies the side of the body with the amputated leg, “+” indicates the normal side of the body, and “;” represents a combination of two amputated legs on different sides. This study specifically examines the consequences of unilateral (± ML ‐ right midleg) and bilateral (‐/‐ ML ‐ midleg pair) amputations.

## Results

### Curve striding in opposite of straight striding

**Fig. 2:**
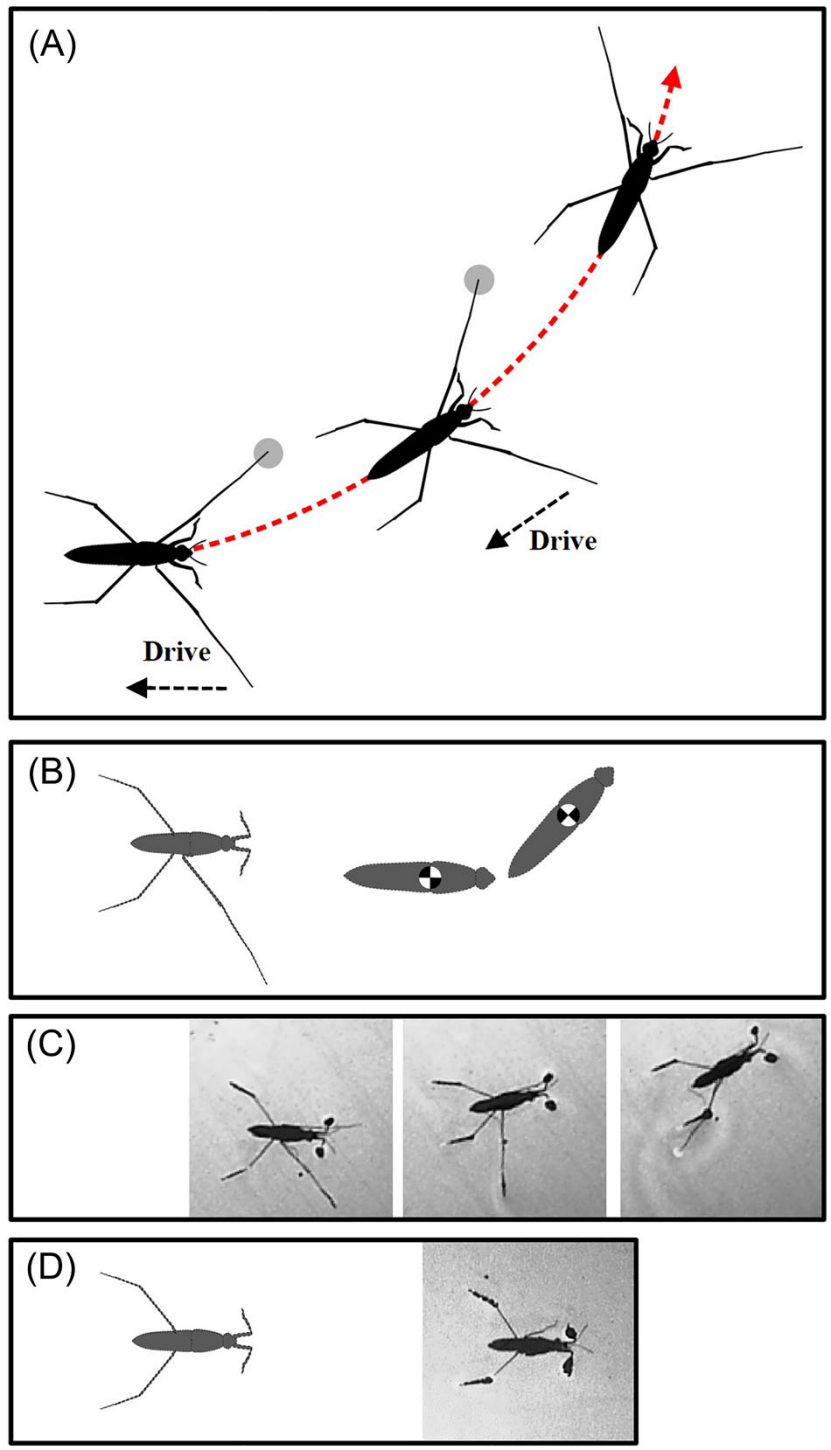
Curve striding. (A) Schematic of curve striding, red dotted‐arrow line shows the body trajectory, gray circles indicate the midlegs contacting the water surface, dashed‐arrow lines indicate midleg moving backward. (B, C) Scheme of an individual with the single midleg (B), and rotation of its body in the set of single video frames (C). (D) Scheme of an individual without both midlegs (left), and its body posture in a single video frame (right).

In typical striding behavior observed in intact *Gerris argentatus*, bilateral symmetric leg movements of all legs play a crucial role in both propulsion and body support (Fig. 1C–F; Video S1). A common locomotion behavior observed in intact water striders is curve striding (Fig. 2A; Video S2).

This turning method optimizes mechanical efficiency by engaging only one midleg in sculling, performing one to three strokes per cycle. The opposite midleg remains stationary, keeping constant contact with the water. Each stroke generates a turning angle of under 40°, but the total rotation can exceed 90°, highlighting the cumulative impact of repeated strokes. Achieving this rotation requires around 40 mm of travel, demonstrating the precision and control involved. This technique, known as curve striding, mirrors the timing of sculling events but focuses on the active midleg, blending rotational dynamics with linear movement to enhance turning efficiency while ensuring stability.

When one midleg is absent, the body rotates approximately 60° toward the legless side at the onset of motion (Fig. 2B). Individuals are unable to row and turn effectively after the loss of two midlegs (Fig. 2C).

### Multiangle turning modes

In all turning maneuvers, the midleg on the opposite side of the turning direction is responsible for generating the striding stroke. At the beginning of a turn, the body adjusts similarly to the starting posture of straight striding, but it transitions into specialized patterns as the turn progresses. Highlighting the complexity of the transition from straight striding to turning, the previously bilaterally symmetric leg movements break down as the turn initiates, resulting in fewer contact points. The remaining legs play a crucial role in supporting the body and adjusting to smooth the turning process. The turn is completed when both midlegs return to their recovery points, reestablishing the starting posture. The number of contact points is a critical factor for maintaining stability during the primary leg movements phase, involving a sequence of kinematic events orchestrated to ensure that five legs are permanently on the water surface. However, during the secondary phase of leg movements, the number of contact points can decline to four, aiding in smoothing the turning process.

**Fig. 3:**
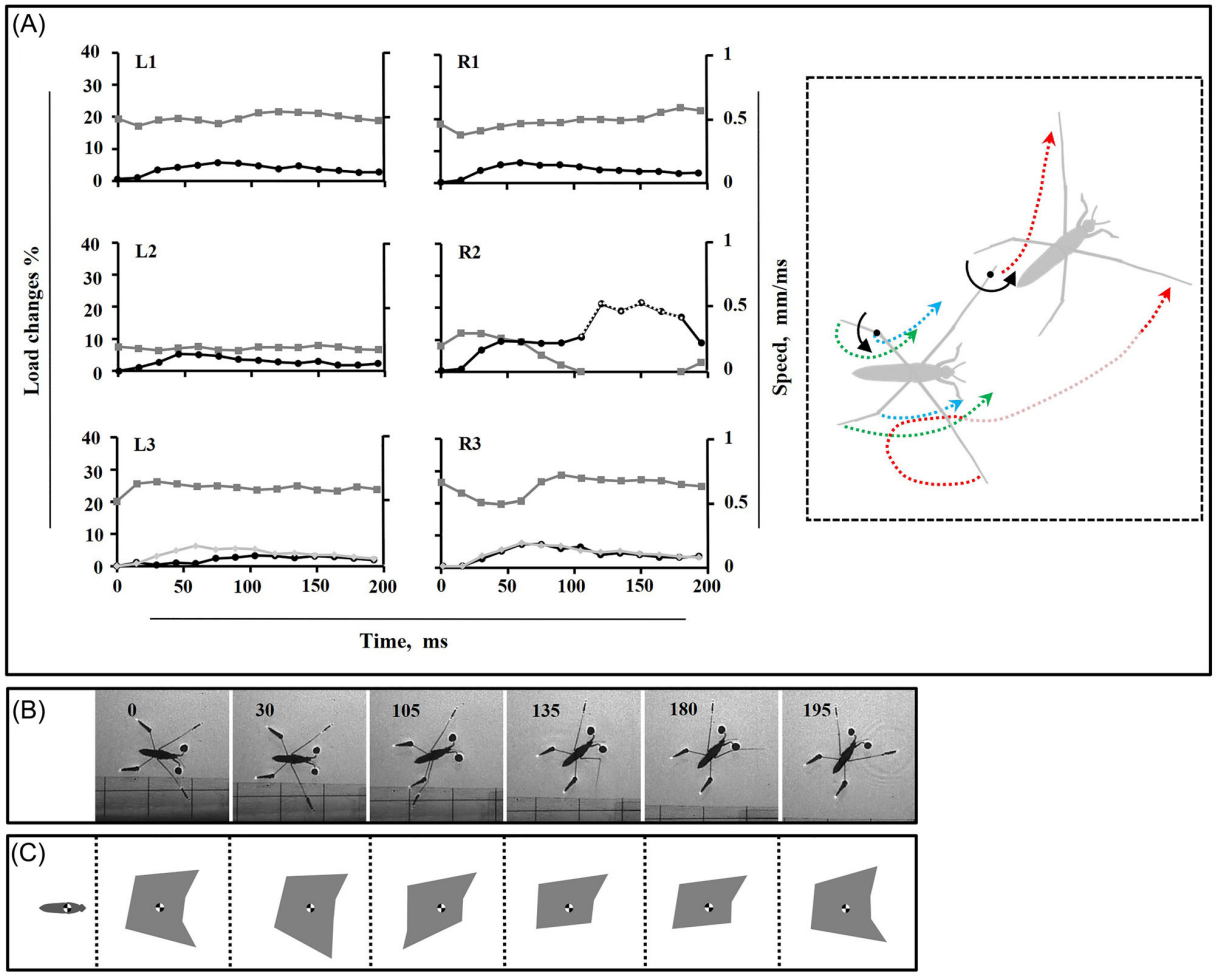
Leg movements during narrow turn. (A) Load distribution on the legs (gray lines), speed of the legs (black lines) along motion trajectory, and the speed of the hindleg tip (light gray), the dotted part of black line for R2 indicates the duration of the midleg in the air, the trajectory of legs during motion. The trajectory of the midlegs (red dotted lines), along with that of the hindlegs including the tips (green dotted lines) and the tibia‐femur joints (blue dotted lines) is depicted during motion in subplot (A). The faint segment of the red dotted lines indicates the duration of the midleg in the air. The curve arrow lines, and the black spots indicate the rotation direction and the points of rotation, respectively. (B) High‐speed sequence of the turn. The number in each frame indicates the time in millisecond. (C) Schemes of corresponding BOS (gray area) during motion presented in (B).

**Fig. 4:**
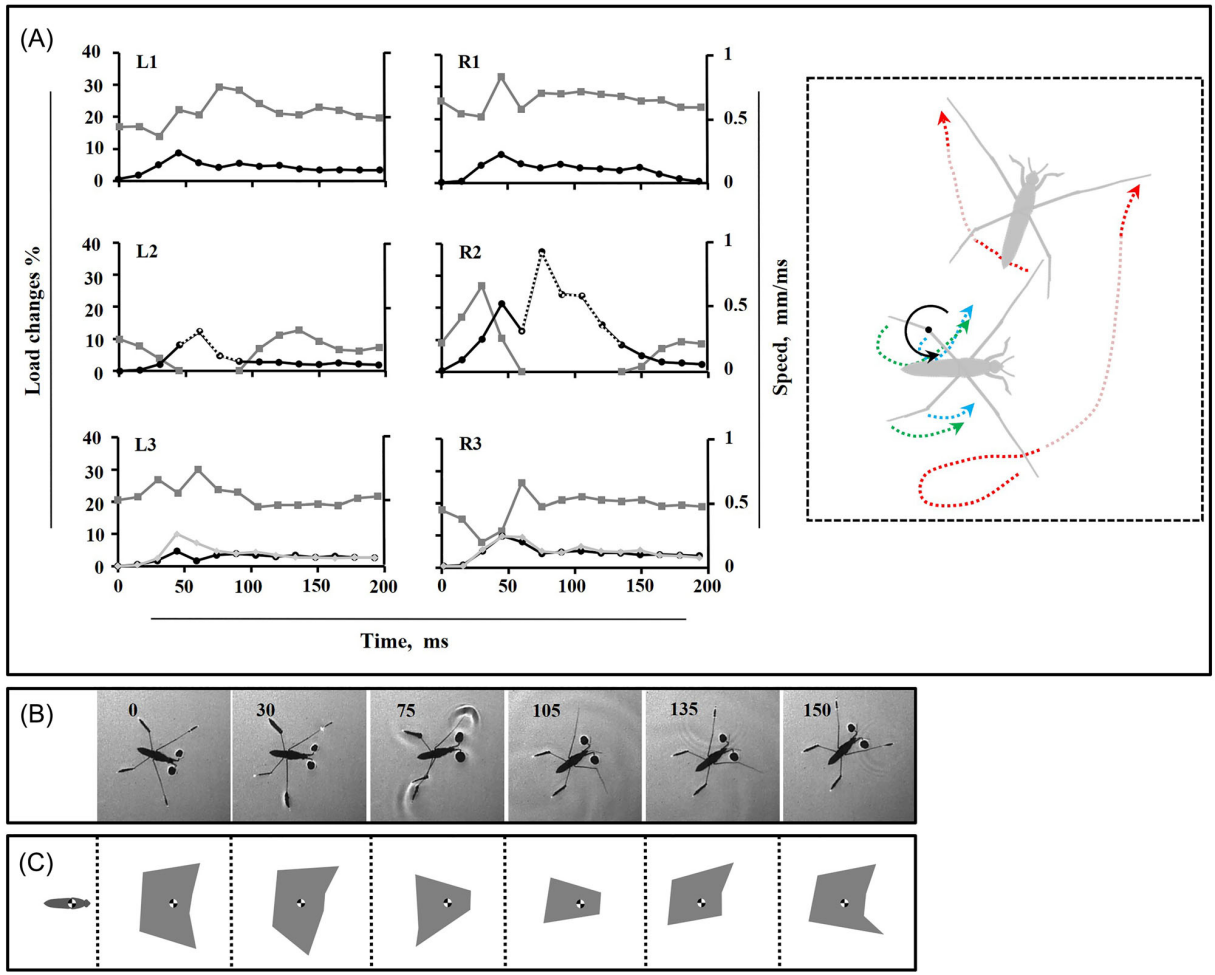
Leg movements during moderate turn. (A) Load distribution on the legs (gray lines), speed of the legs (black lines) along motion trajectory, and the speed of the hindleg tip (light gray), the dotted parts of black lines for L2 and R2 indicate the duration of the midleg in the air, the trajectory of legs during motion. The trajectory of the midlegs (red dotted lines), along with that of the hindlegs including the tips (green dotted lines) and the tibia‐femur joints (blue dotted lines) is depicted during motion in subplot (A). The faint segments of the red dotted lines indicate the duration of the midleg in the air. The curve arrow line, and the black spot indicate the rotation direction and the point of rotation, respectively. (B) High‐speed sequence of the turn. The number in each frame indicates the time in millisecond. (C) The adjusted schemes within the straight line of the corresponding BOS (gray area) during motion presented in (B).

**Fig. 5:**
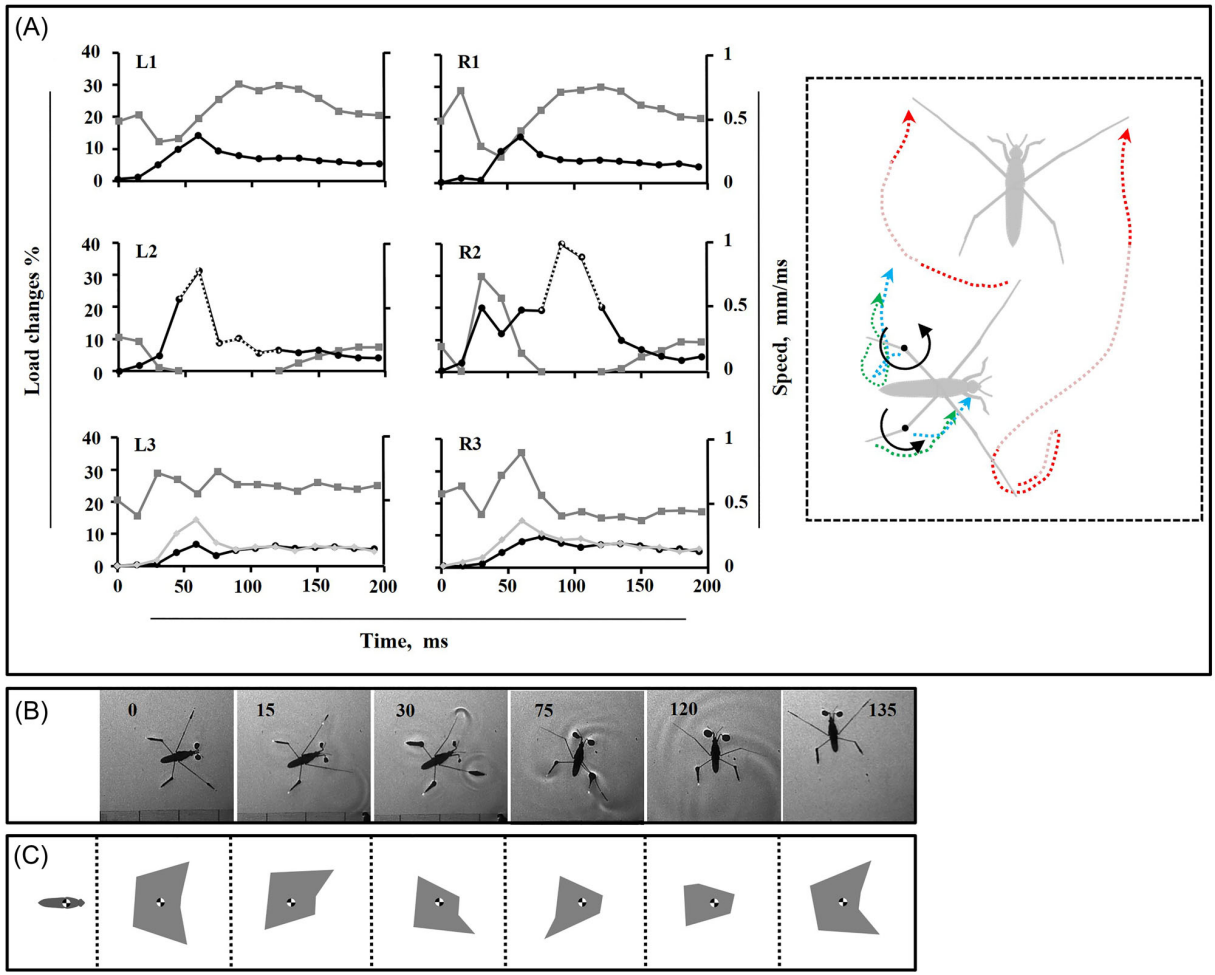
Leg movements during wide turn. (A) Load distribution on the legs (gray lines), speed of the legs (black lines) along motion trajectory, and the speed of the hindleg tip (light gray), the dotted parts of black lines for L2 and R2 indicate the duration of the midleg in the air, the trajectory of legs during motion. The trajectory of the midlegs (red dotted lines), along with that of the hindlegs including the tips (green dotted lines) and the tibia‐femur joints (blue dotted lines) is depicted during motion in subplot (A). The faint segments of the red dotted lines indicate the duration of the midleg in the air. The curve arrow lines, and the black spots indicate the rotation direction and the points of rotation, respectively. (B) High‐speed sequence of a turn. The number in each frame indicates the time in millisecond. (C) The adjusted schemes within the straight line of the corresponding BOS (gray area) during motion presented in (B).

A turning is considered complete when both midlegs reach the recovery points, although in some cases, the body may slide to widen the turn angle. The final headings achieved by the studied group of water striders varied in angle degrees, leading us to categorize them into three levels: narrow turning (*θ* < 60°), moderate turning (*θ* = 60°−80°), and wide turning (*θ* > 80°) (Figs. 3, 4, 5) (Videos S3, S4, S5). Based on our observations, the times required for narrow, moderate, and wide turns are 192 ± 5, 150 ± 8, and 135 ± 6 ms, respectively. Similar to how the body continues to slide after completing the sculling stroke during straight striding, in each turning mode, the body also experiences a slight sliding motion, which may result in a wider turning angle.

In narrow turns, the nonsculling midleg serves as a pivotal anchor, allowing the body to rotate smoothly along a curved path. This fixed position of leg creates a stable axis for rotation. In moderate and wide turns, while the same rotational mechanism is used, the midleg often loses contact with the water, reducing its influence on pivoting and requiring greater reliance on other forces and legs to maintain stability and control the turn. This shift in dynamics is especially pronounced in wide turns.

### Leg placement and trajectory during turning maneuvers

Our observations reveal that turning motion in the experimental hexapod animal involves significant changes in leg alignments and body posture. The irregular sequence of leg movements contributes to the observed fluctuations in instant load distribution, as depicted in the graphs (Figs. 3, 4, and 5A). Therefore, we believe that measuring speed alone is insufficient for assessing leg function, and load changes on the legs should also be considered. This approach allows us to determine the center of rotation and pivot points, around which the body twists, while simultaneously sliding on the water surface. We plotted the instant speed and load changes during turning trials on separate graphs (Figs. 1, 3, 4A, and 5A).

Because of symmetrical leg kinematics in intact water striders, we utilized speed and load change graphs from one side as an indicator for comparing different turning modes (Fig. 1C). As midleg motion speed increases, the range of body rotation expands, transitioning from narrow angles at slower speeds to moderate and eventually wide angles at higher speeds (Figs. 3, 4, 5). During each level of turning, both the forelegs and hindlegs frequently realign, with the tarsus‐tibia sections of the hindlegs on both sides rotating inward relative to the femur, especially as speed changes become more pronounced.

During narrow turns, one midleg remains on the water surface while the other exhibits typical sculling movements, including drive, swing, and recovery stages (Figs. 1F and 3B). Once the recovery phase of the sculling midleg ends, the body motion is completed, though sliding continues for milliseconds until a full stop occurs. For moderate turning, despite sculling by one midleg, both midlegs detach from the water surface with distinct timing (Fig. 4). In the middle of the sculling stroke, one midleg is raised while the sculling midleg drives backward. The nonsculling midleg does not swing but relocates to a new position concurrently with the swinging of the sculling midleg, restricting the rotational motion of the body during narrow turns. However, this effect is mitigated by suspending the nonsculling midleg in the air as the degree of rotation increases.

Wide turning is similarly achieved by detaching both midlegs from the water surface, but with staggered timing (Fig. 5). The midleg on the turning side detaches 30 ms earlier than the opposite side. The striding stroke process resembles typical sculling, with the responsible midleg initially swinging forward to a new catch point and then moving backward to the finish point. The sculling midlegs drive a longer distance than usual, while the opposite midleg remains suspended longer than in moderate mode.

### Widening of sculling field and reverse sculling

**Fig. 6:**
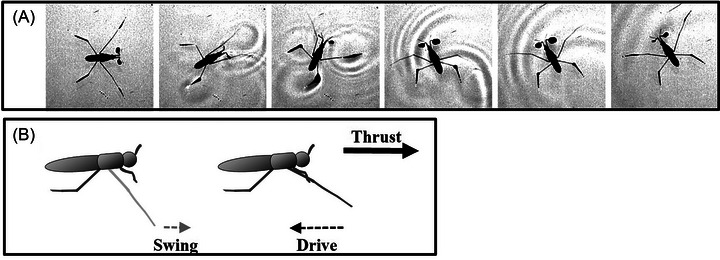
Excessively wide turn. (A) Sequences of an excessively wide turn. (B) Schematic of further swinging of the midlegs during the wide turn.

At the starting posture, the femur and body centerline form an angle of approximately 56° between each midleg (Meshkani *et al.*, 2023a). In the wide turning mode, the midleg swings forward slightly so that the angle between the midleg and the body centerline decreases to 35° (Fig. 5B), which allows the midleg to drive wider (Video S6). For an excessively wide turning, individuals can swing their midlegs highly forward, reducing the midleg angle to as low as 10°, as observed (Fig. 6A, B).

**Fig. 7:**
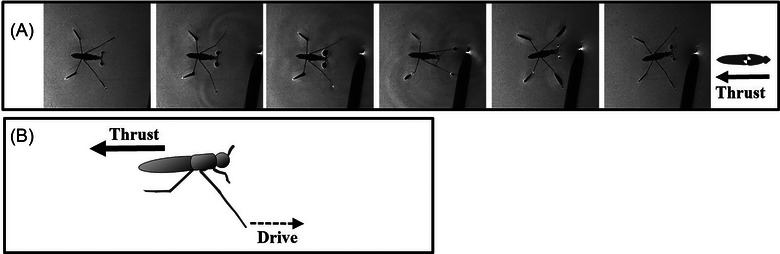
Reverse sculling. (A) High speed sequence of the reverse sculling. (B) Schematic of reverse sculling of the midlegs

It is worth noting that water striders can execute reverse sculling when confronted with a threatening object (Fig. 7A, B) (Video S7). In this case, water striders perform sculling while the midlegs move forward to propel the body backward. The body slides slowly for approximately 22 mm during reverse sculling, which lasts about 90 ms. In comparison, normal sculling takes around 75−90 ms, allowing the body to slide for about 715 ms and cover a greater distance of 38 mm.

**Video S1:** Video clip of striding.

**Video S2:** Video clip of sculling (with an amputated midleg).

**Video S3:** Video clip of the narrow turning.

**Video S4:** Video clip of the moderate turning.

**Video S5:** Video clip of the wide turning.

**Video S6:** Video clip of the excessively wide turning.

**Video S7:** Video clip of the reverse sculling.

## Discussion

### Leg specialization effects on maneuverability

Water striders have evolved highly specialized legs that can be classified into two functional categories: scull‐legs, represented by the midlegs, and ski‐legs, which combine the forelegs and hindlegs. During striding, the scull‐legs execute strokes that facilitate the sliding motion of the ski‐legs. The midlegs initiate movement by having their tips travel from the catch point, where they first contact the water surface, driving backward, and then lifting and detaching at the finish point (Fig. 1F). Each midleg subsequently swings through the air to reach the recovery point in the anterior position. The movement of the legs is synchronized with changes in load on the legs, which is crucial for stable and efficient locomotion on water (Fig. 1C) (Lu *et al.*, 2018; Meshkani *et al.*, 2023a). While the midlegs primarily bear the dynamic load for propulsion, the forelegs and hindlegs focus on stabilization, preventing disproportionate variations that could disrupt the movement of water striders (Steinmann *et al.*, 2018; Meshkani *et al.*, 2023b).

Water striders can seamlessly transition between straightforward striding and curve striding, allowing for smooth changes in gait and direction, which enhances their maneuverability as they explore their environment. To maintain balance during these directional changes, they shift into specialized leg movement patterns as the turn progresses. These patterns become increasingly complex, with irregular sculling as they transition from narrow angles at slower speeds to moderate and wide angles at higher speeds. By effectively combining straight and curve striding, water striders minimize the risk of losing stability during these maneuvers. During curve striding, the body pulls to the side of the midleg attached to the water surface, which serves as a pivot, resulting in the body moving along an arc trajectory. Pivot points endure loads, while other components revolve around them.

The amputation of midlegs significantly affects the striding trajectory, starting with alterations in body adjustment during the starting posture (Meshkani *et al.*, 2023b). This disruption leads to changes in motion characteristics, including a notably higher rotation angle of the body that results in the arc trajectory (Fig. 2B, C). The comparison of intact and amputated individuals suggests that the motionless midleg limits the degree of rotation, highlighting the crucial role of midlegs for directing the body during turning.

After losing a leg, the remaining legs support the body to maintain maneuverability (Goetz & Wenking, 1973). The imbalanced load distribution resulting from the loss of any ski‐legs causes the body to bend toward the side with higher load bearing, leading to skidding and deviation from the desired trajectory (Meshkani *et al.*, 2023b). This effect emphasizes the importance of the ski‐legs in fine control of body balance and locomotion trajectory. Previous studies have shown that forelegs and hindlegs do not contribute to sculling strokes (Lu *et al.*, 2018; Meshkani *et al.*, 2023b), and observations on amputees without both midlegs indicated that forelegs and hindlegs are also not involved in the start of turning (Fig. 2D). In contrast, walking insects utilize multiple legs for turning (Dean & Wenler, 1982; Camhi & Levy, 1988; Jindrich & Full, 1999; Wosnitza *et al.*, 2013). This suggests a clear distinction between the specialized roles of water strider legs for sculling and turning, compared to the more generalized leg use in walking insects.

### Leg placement affects turning

The purpose of turning in water striders is to alter their motion from an initial direction to a new one through a range of coordinated leg movements. Water striders exhibit various patterns of leg coordination depending on the desired type of motion, similar to walking insects (Grabowska *et al.*, 2012; Han *et al.*, 2024). Notably, water striders complete turning maneuvers faster for wider angles, which may serve as a strategy to produce stronger power and reduce the amount of time the body spends in an unrestrained state (Figs. 4A and 5A).

During each level of turning, both the forelegs and hindlegs frequently realign. Notably, the tarsus‐tibia sections of the hindlegs rotate inward relative to the femur, aligning nearly parallel to the body axis (Figs. 3, 4, and 5B). This inward rotation is likely linked to the stabilization role played by the forelegs and hindlegs during the turning process.

In walking insects, the forelegs guide the body toward the desired direction while other legs actively contribute to turning (Camhi & Levy, 1988; Dürr & Ebeling, 2005). For example, in honeybees, synchronized movements of forelegs accompany gait transition and guide turning direction (Zhao *et al.*, 2018). However, in water striders, we found that the initiation and direction of turning are primarily controlled by the midlegs. Suspending a nonsculling middle leg may facilitate body turning, enabling a wide range of maneuvers. Conversely, other legs might play a role in restricting turning to some extent. We predict that this coordinated action of the legs allows for precise turning behavior depending on the situation.

### Dynamics of turning modes

Despite uneven leg pressure points, insects have evolved a remarkable ability to balance their weight on the water surface (Zheng *et al.*, 2016). Stabilization of a floating body is regulated continuously by leg adjustments and the compensatory role of legs relative to each other (Lu *et al.*, 2018; Meshkani *et al.*, 2023a). A series of leg movements is necessary to control turning motion in walking insects (Dürr & Ebeling, 2005), and our observations reveal that turning motion in water striders involves substantial changes in leg alignments and body posture. Assessing leg function using load changes and speed allows us to determine the center of rotation and pivot points in the experimental hexapod animal (Figs. 3, 4, and 5A). Turning involves the body twisting around these pivot points, while simultaneously the whole body, including the pivot points, slides on the water surface. In narrow turns, the nonsculling midleg serves as a pivot point, allowing the body to slide along a broad curve and rotate around that leg. Moreover, the variation in speed indicates an adjustment in the movement of the hindleg tip, involving an inward and outward shift on the nonsculling midleg side. This suggests that the tibia‐femur joint of the hindleg on the nonsculling side functions as a pivot point, at least temporarily (Fig. 3A). Simultaneously, the reduction of load on the opposite hindleg facilitates rotation, likely by reducing the drag resistance.

This rotational model is repeated for moderate and wide turns, with the exception that the nonsculling midleg has no contact with the water surface and may not be considered a pivot (Figs. 4A and 5A). Instead, the rotational center shifts to the tibia‐femur section of the hindleg, as well as the second pivot on the side of the sculling midleg. As the degree of turning increases, the rotation around the primary rotational points becomes more pronounced. In the case of wide turns, an additional rotational point with a smaller degree of rotation emerges on the opposite side of the turn. Therefore, the body has an opportunity to twist toward the turning side while the nonsculling midleg is suspended in the air. This situation is more amplified for wide turns (Fig. 5A), especially since it coincides with a longer duration of the drive stage. The graphs also depict an irregular distribution of instant loads due to this irregular sequence of leg movements. The turnings may be influenced by additional controlling factors affecting the freedom of the body rotational degree.

Following a wide turn, the increased load on the forelegs suggests the body tilts anteriorly (Fig. 5A, B), which could consequently elevate the risk of falling and becoming trapped at the water surface. While the flexibility of their legs helps water striders conform to the water surface, enhancing stability and sculling effectiveness (Wei *et al.*, 2009; Ji *et al.*, 2012; Koh *et al.*, 2015; Steinmann *et al.*, 2018; Yan *et al.*, 2018), these insects appear to employ a rapid recovery strategy to minimize time spent in unstable positions and prevent tipping over. Obtaining further evidence through additional investigations would strengthen the link between leg flexibility and successful turning maneuvers in water striders.

### Kinematics of legs throughout a turn

Water striders maintain stability on water with six points of contact, positioning their body center over the middle of the base of support (BOS) (Fig. 1D, E). The BOS refers to the area encircled by the perimeter connecting points of leg contacts with the water. Studying the BOS provides insight into potential challenges related to stability loss during locomotion (Hughes, 1952). The symmetrical BOS shape is crucial for maintaining postural equilibrium (Binder *et al.*, 2008) and preventing the wastage of stroke power during movement. Whenever a leg contact is missed, a new BOS shape is established, differing in size and shape from the regular symmetric BOS shape (Meshkani *et al.*, 2023b). Instability increases when the body center is situated near or outside the regular BOS configuration, as observed in water striders with amputated legs (Meshkani *et al.*, 2023b).

In our study, we assessed the re‐establishment of the BOS during each turning mode (Figs. 3, 4, and 5C), observing various BOS configurations throughout each stage of motion. Most changes in the BOS are attributed to the movements of midlegs on the surface or their motion in the air. Despite the ski‐legs remaining attached to the water surface, their realignment was evidently effective in improving stability. Within each stage of motion, the ski‐legs exhibited frequent accelerations and decelerations to achieve a proper BOS shape (Figs. 3, 4, and 5A). During the primary leg movements phase, a minimum of five contact points is necessary to maintain an asymmetrical BOS. However, during the secondary phase, the number of contact points can decline to four, while the BOS maintains a mostly symmetrical shape. This finding contrasts with previous studies, such as Zhao *et al.* (2018), which indicated that honeybees use the tetrapod gait to achieve turning.

As water striders execute a rapid wide turn, the body enters a state of pronounced misalignment, giving the appearance of being momentarily unrestrained. Consequently, BOS remodeling becomes highly irregular compared to forward striding. To maintain smooth turning, the ski‐legs, particularly the hindlegs, actively contribute to re‐establishing BOS through the rotation of their joints. Thus, the dynamic adjustments of the ski‐legs are critical for the ability of water strider to execute, and control turns efficiently.

### Adaptation of legs for unexpected movements

In insects, joint articulations restrict the movements of leg segments to specific planes and to specific opening angles (Hoyle, 1976; Delcomyn, 1981; Frantsevich *et al.*, 1996; Frantsevich, 1998; Frantsevich & Gorb, 2004). Despite limited knowledge about leg kinematics in water striders, our results suggest that their legs exhibit a high degree of flexibility and range of motion.

We observed that the midlegs swing farther from the catch point, extending anterior to the body, which enables the insect to scull at a wider angle (Figs. 6A, B). This extended motion increases both the length and duration of the midleg drive, allowing the body to achieve a greater degree of rotation through prolonged sculling. The increased range and duration of this movement enhances maneuverability and speed during wide‐angle turns.

Reverse sculling, compared to regular straightforward sculling, demonstrates a clear inefficiency in terms of length and duration. Despite this inefficiency, our observations strongly indicate that the muscular system and joint articulations of the midlegs are adapted to propel themselves backward (Figs. 7A, B). This adaptation may provide water striders with a unique escape maneuver, especially in situations where the risk associated with turning or moving forward is high.

## Conclusion

Changing the direction of body motion is a dynamic process that can lead to instability. Asymmetrical load distribution during body rotation increases energy expenditure during motion (Perez Goodwyn *et al.*, 2009). In water striders, turning is not a sudden action but involves a series of coordinated leg movements, including accelerations and decelerations. This behavioral transition requires a specific orchestration of events across all legs, each playing distinct roles as the body shifts from its initial heading to its final heading. Turning is initiated and guided by one midleg, with ski‐legs contributing to smooth transitions. The speed of midleg motion dictates the body rotation, with slower movements causing minimal rotation and faster movements inducing larger rotations. Although ski‐legs do not generate power strokes, they are crucial by providing pivot points, remodeling the base of support (BOS), and altering drag at contact points with the water surface. Primarily, hindlegs serve as pivot points in moderate to wide turns. Turning in water striders involves a combination of body rotation and sliding, with a higher rate of body rotation observed in moderate and wide turns. They utilize a faster body rotation rate to minimize periods of dynamic instability during wide turns, allowing them to quickly realign and prevent toppling. There is a correlation between adjustments in the spatiotemporal configuration of leg joints and turning movements. Water striders possess a unique adaptation in their midlegs for backward locomotion. The muscular system and joint articulations of these legs are specifically designed for propelling the insect backward, showcasing a high degree of flexibility and range of motion. Understanding how water striders change their body motion direction can potentially simplify the control of maneuvers for walking robots under specific locomotory demands and conditions, such as striding on the water surface (Billeschou *et al.*, 2021; Larsen *et al.*, 2023; Phodapol *et al.*, 2023).

## Disclosure

The authors declare there are no conflicts of interest to disclose.

## Data accessibility

All supporting data are made available in the article.
